# Internal cueing improves gait more than external cueing in healthy adults and people with Parkinson disease

**DOI:** 10.1038/s41598-018-33942-6

**Published:** 2018-10-19

**Authors:** Elinor C. Harrison, Adam P. Horin, Gammon M. Earhart

**Affiliations:** 1 Program in Physical Therapy, Washington University School of Medicine, St Louis, Missouri United States of America; 2 Department of Neurology, Washington University School of Medicine, St Louis, Missouri United States of America; 3 Department of Neuroscience, Washington University School of Medicine, St Louis, Missouri United States of America

## Abstract

Walking can be challenging for aging individuals and people with neurological disorders such as Parkinson disease (PD). Gait impairment characterized by reduced speed and higher variability destabilizes gait and increases the risk of falls. External auditory cueing provides an effective strategy to improve gait, as matching footfalls to rhythms typically increases gait speed and elicits larger steps, but the need to synchronize to an outside source often has a detrimental effect on gait variability. Internal cueing in the form of singing may provide an alternative to conventional gait therapy. In the present study, we compare the effects of internal and external cueing techniques on forward and backward walking for both people with PD and healthy controls. Results indicate that internal cueing was associated with improvements in gait velocity, cadence, and stride length in the backward direction, and reduced variability in both forward and backward walking. In comparison, external cueing was associated with minimal improvement in gait characteristics and a decline in gait stability. People with gait impairment due to aging or neurological decline may benefit more from internal cueing techniques such as singing as compared to external cueing techniques.

## Introduction

Age-related gait disorders affect a third of the population over 70 years of age^1^ and cause people to walk slower with less stability. Reduced gait speed in older adults is a sensitive marker of overall health and can predict adverse events, such as falls, and future disability^2,3^. Two-thirds of gait disorders are related to neurological decline^4^ and are exacerbated in movement disorders such as Parkinson disease (PD). PD is characterized by bradykinesia, rigidity, and postural instability, all of which contribute to walking difficulty^5^. Compared to age-matched controls, people with PD experience accelerated gait decline as evidenced by reductions in speed, step frequency, and step length. In addition to these basic gait deficits, people with PD exhibit substantial increases in gait variability^6^ which may reflect diminished balance control^7^ and a disruption of internal timing mechanisms within the brain. Gait variability is a strong indicator of overall stability^8–10^, worsens with disease severity, and may lead to a loss of mobility and independence^11,12^. When moving in the backward direction, as is common in everyday life, gait impairment is more pronounced and more likely to contribute to fall risk^13–15^. Hence, a major focus of gait therapy is to reduce gait variability in order to stabilize walking and reduce the risk of falls.

External auditory cueing through music is widely established as an effective tool to normalize gait disturbance^16–18^. For people with PD, matching one’s footfalls to the beat of a song can restore gait to levels closer to those of healthy controls^16,17,19,20^. Rhythmic cues allow predictable mapping of motor output onto stable auditory templates via a process called “entrainment” that enables people to anticipate the next beat and step on it. Musical cues are superior to other types of cues at increasing velocity and stride length^19^ though they are more effective after a period of training^21^ and for those with more severe gait impairment^22^.

In spite of evidence supporting the efficacy of rhythmic auditory cues for improving certain gait characteristics^17,23–26^, recent research suggests that synchronizing footfalls to external rhythmic cues detrimentally effects gait variability^27^. External cues require adjusting every step in order to synchronize, and this increased cognitive load may have the undesirable effect of increasing gait variability, particularly for older adults or neurological patients who are more likely to experience cognitive decline^28^. Internal cueing through singing, on the other hand, eliminates the need to entrain to an external source. Instead, a rhythm generated and produced via the vocal system is then adopted by the locomotor system to produce rhythmic motion of the legs. This method may allow for greater coupling between systems, potentially reducing attentional load and enhancing stability.

Singing is already used for vocal rehabilitation in PD because, in spite of speech degradation, singing ability is preserved^29–31^. Evidence also suggests that the benefits of singing may extend beyond speech to improvements in motor control^32^ as singing may engage a vocal sensorimotor loop involving both perceptual and motor planning components^33^. For example, self-generated vocal cues enhance upper extremity movement in people with PD, resulting in faster and smoother reaching movements^34^. Vocalizations are also likely to enhance lower body movement, as people with PD report using singing to aid with gait initiation and maintenance, particularly during challenging gait situations such as moving backwards and turning^35^. Despite abundant evidence supporting the use of singing to improve walking in aging and neurologic populations, previous research is mostly limited to the use of external cueing for gait rehabilitation.

In this study, we examined the effects of internal cueing, in the form of singing, versus external cueing, in the form of listening to music, on gait in people with and without PD. We addressed both forward walking, which engages automatic locomotor circuits, and backward walking, which represents a more challenging gait situation. We hypothesized that both external and internal musical cueing would improve backward walking more than forward walking in all our participants, and that internal cueing would be more effective at reducing gait variability over external cueing. We also expected to see the greatest benefit from cueing in people with PD, followed by older adults and finally younger adults.

## Results

### Gait characteristics

#### Differences between conditions

**Figure 1:**
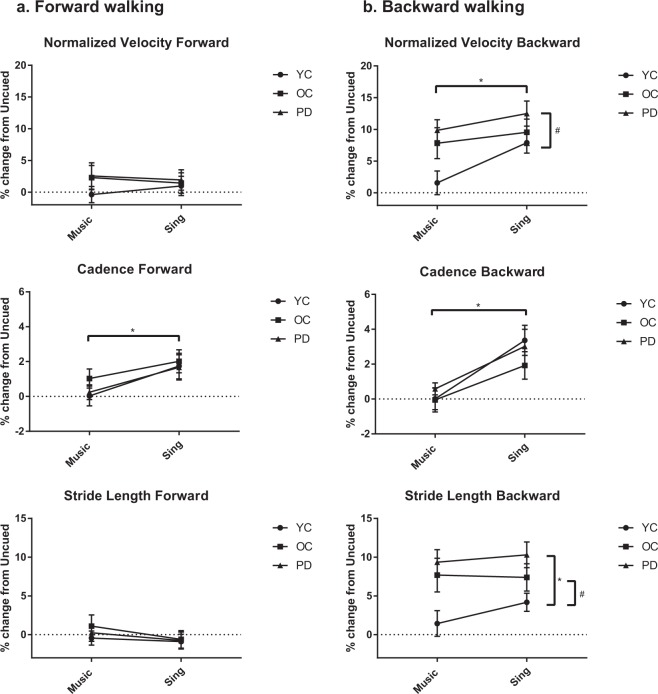
Gait characteristics shown as a percent change from Uncued walking compared across groups for forward and backward walking. All bars represent means ± SEM. Horizontal significance bars indicate an overall effect of condition, whereas vertical significance bars indicate an overall effect of group. *Indicates p < 0.01. ^#^Indicates p < 0.05.

In forward walking, there was an overall effect of condition (F(1, 87) = 6.978, p < 0.001) with univariate tests showing a significant increase in cadence for SING versus MUSIC (F(1, 87) = 15.121, p < 0.001). (Fig. 1, Supplementary Table 1).

In backward walking, there was an overall effect of condition (F(1, 87) = 8.396, p < 0.001) with univariate tests showing that participants walked faster (F(1, 87) = 10.868, p = 0.001) with higher cadence (F(1, 87) = 22.523, p < 0.001) in SING as compared to MUSIC.

#### Differences between groups

There were no significant differences between groups in forward walking gait characteristics.

In backward walking, there was a significant between-subject effect of group for velocity (F(2, 87) = 3.552, p = 0.033) and stride length (F(2, 87) = 5.744, p = 0.005). Regardless of condition, pairwise comparisons indicated that the PD group showed a more robust response to cueing than the YC group as evidenced by their greater percent change in velocity (p = 0.010) and their greater percent change in stride length (p = 0.001). The OC group also showed a greater percent change in stride length as compared to the YC group (p = 0.028). There were no significant interactions, indicating that all groups responded similarly to cueing.

### Gait variability

#### Differences between conditions

**Figure 2:**
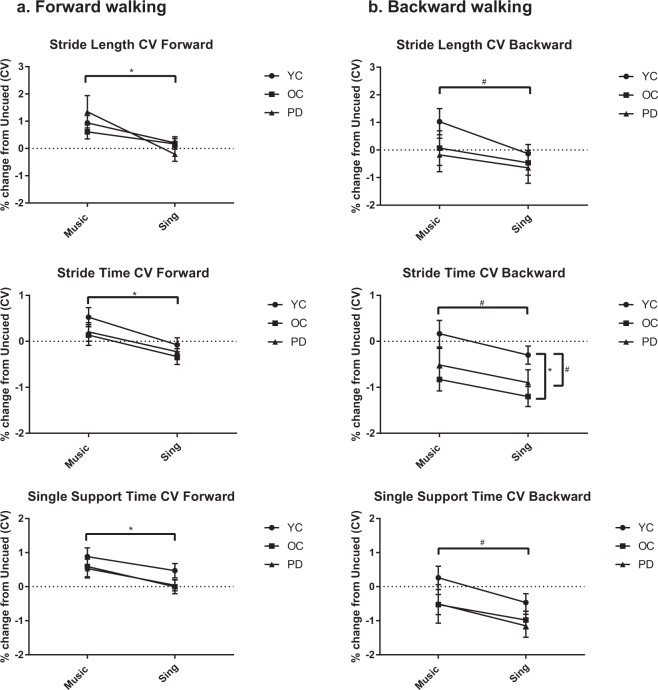
Coefficients of variation compared across groups for forward and backward walking. All bars represent means ± SEM. Horizontal significance bars indicate an overall effect of condition, whereas vertical significance bars indicate an overall effect of group. *Indicates p < 0.01. ^#^Indicates p < 0.05.

In forward walking, all participants walked with less variability in SING than in MUSIC, as evidenced by a significant main effect of condition (F(1, 87) = 14.564, p < 0.001) (Fig. 2). This was significant for CVs of stride length (F(1, 87) = 20.039, p < 0.001), stride time (F(1, 87) = 27.623, p < 0.001), and single support time (F(1, 87) = 10.673, p = 0.002).

For backward walking, participants walked with less variability in SING than in MUSIC, as there was a main effect of condition (F(1, 87) = 3.035, p = 0.034). This was significant for CVs of stride length (F(1, 87) = 5.498, p = 0.021), stride time (F(1, 87) = 5.793, p = 0.018), and single support time (F(1, 87) = 6.825, p = 0.011).

#### Differences between groups

There were no significant differences between groups in forward walking variability.

In backward walking, there was a significant main effect of group for stride time (F(2, 87) = 4.525, p = 0.014). Pairwise comparisons revealed that the OC group (p = 0.004) and the PD group (p = 0.05) had significantly less variability regardless of condition than the YC group. There were no significant interactions.

## Discussion

In this study, we examined the effects of internal versus external cues on forward and backward walking in three groups of people: healthy young, healthy older, and people with PD. The results support our hypotheses, that internal cueing in the form of singing may be more beneficial to gait than external cueing. The results also confirm that people with PD exhibit greater improvement than their healthy counterparts^36^ and may stand to gain the most benefit from internal cueing techniques, particularly in challenging gait situations such as moving in the backward direction.

One of our primary results was that singing increased cadence in both walking directions. In backward walking, this increase in cadence led to higher velocity as well. External cues, in contrast, did not have a significant effect on gait speed, cadence, or stride length on forward walking and had a lesser effect than internal cues on backward walking. This is in accordance with previous studies of forward walking showing only small effects of external cues at preferred walking tempos^37,38^ and with a recent review revealing generalized small effects on velocity and cadence in cueing without training^20^. During MUSIC, the cadence was set by the cue, and as we explicitly told participants to synchronize to it, they did not stray far from baseline. In SING, by contrast, with no outside source dictating the song tempo, participants tended to increase their cadence as they sang.

One possible explanation for this is that active music-making (such as singing) may confer greater motor benefits than passive music listening^39^ by tapping into reward circuitry and affecting movement “vigor,” both of which are compromised in PD. Endorphin and oxytocin release during singing has positive effects on motivation and may translate into higher motor output^39–42^. Singing is also known to activate motor regions in the brain including the primary motor cortex, the basal ganglia, thalamus, and cerebellum^43,44^, which may additively combine with motor activation during locomotion. While synchronizing movement to music may induce an arousal effect that makes movement faster, larger, and more vigorous^45^, synchronizing movement to one’s own voice may lead to even greater overall motor network activation and, hence, higher cadence^46^.

We also noted that, in relation to baseline, external cues had a detrimental effect on forward-walking variability. This supports previous work showing that, for healthy young adults with low baseline variability, external cues tend to perturb normally-functioning internal cueing mechanisms and interfere with gait stability^47–50^. Similarly, older adults do not benefit when constrained by external cues, as gait variability is either unaffected^19^ or increased with cues at preferred cadence^48,50,51^. Cues at tempos below^52^ or above^36^ preferred cadence also increase gait variability^24,48^.

For people with PD, preferred cadence cues have shown no effect^24,51^ or increases in variability^52^, even after training^22^. Reductions in variability have been reported, but only for faster tempos and after a brief period of training^53^. The sum of these studies shows that isochronous external cues lend only a minor benefit to gait characteristics and may come at the price of sacrificing temporal stability, particularly for those with more impaired baseline gait.

In contrast, singing did not negatively affect gait variability. In forward walking, internal cues did not cause gait decrement, and in backward walking, internal cues elicited greater reductions in variability than external cues. The effectiveness of internal cues over external cues in decreasing gait variability may be partially explained through several speculations detailed below.

While external rhythms rely on auditory-motor coupling within the brain to perceive sensory stimuli and match body movement to them, internal rhythms utilize what we will refer to as *vocal-motor coupling*. As humans are capable of entrainment within both the vocal and motor systems, it is possible that matching one system’s output to that of another through self-generated cues allows for greater stability. Entrainment of one system to another within the same body may reduce attentional load and facilitate motor synchronization. Additively combining motor output from two effectors within one individual may reduce variability in a central timing process that results in lower movement variability. For instance, a bimanual advantage makes tapping with two hands less variable than tapping with only one^54^.

A similar mechanism may be at play when a motor effector matches a vocal effector. Skills in motor synchronization and singing are strongly linked, as the neuronal networks that support sensorimotor translation in both partly overlap^55^. Aligning speech to movement enhances verbal processing and facilitates temporal predictions, as information at expected times is processed more efficiently^56^. Furthermore, concurrent rhythmic vocalizations can reduce variability of whole-body movement, which suggests that moving and vocalizing as a coordinative structure causes mutual stabilization between systems^57^. As seen through the lens of an internal model, feedforward control during singing masks auditory feedback and allows singers to continuously phonate without processing each note before continuing. By canceling out reafferent signals to the auditory cortex, singing may reduce reliance on real-time auditory feedback that is necessary with external cues, thereby increasing predictability and decreasing motor variability^58^.

Better synchronization when singing may also be related to our bias for hearing the human voice, or a “vocal advantage”. This postulates that it is easier to match stimuli to personal motor representations that are recognized as biologically possible. The voice is a highly salient stimulus that causes enhanced arousal^59^, greater pupil dilation^60^, and greater activation in the sensorimotor cortex^61^ in listeners as compared to non-vocal melody perception. The dorsal auditory stream, which connects the auditory and motor cortices, has stronger connectivity when participants listen to singing-voice versus non-vocal music, facilitating matching between perceived sounds and motor representations^62^, and sung melodies are better encoded than instrumental melodies, resulting in faster auditory processing^63^. Faster processing and stronger dorsal stream connectivity may enable motor improvement during vocally-*produced* sounds as well.

Notably, the PD group exhibited the largest response from internal cueing. This implies that, in spite of basal ganglia degeneration linked to internal timing deficiencies^64–67^, people with PD were not only capable of internally generating rhythms through singing but were also able to match their movement to it. Beat impairment in PD is thought to impact movement as specific motor network regions, such as the basal ganglia, cerebellum, premotor cortex, and supplementary motor areas, are also responsible for rhythm processing^16,68^. Neurodegeneration of these motor regions may disrupt the internal regulation of movement amplitude and timing in PD and lead to an inability to control automatic locomotor rhythm^67^. For people with PD, for whom disease-related decreases in striatal dopamine affect excitatory input to the putamen, external cues are thought to reduce reliance on putamen activity by compensating for impaired internal timing mechanisms^69^. Singing may achieve the same end by rerouting temporal sequencing from the impaired basal-ganglia-thalamocortical network to other brain areas, such as the spared cerebellar-thalamocortical network, which regulates perceptual and motor timing, or the premotor cortex (PMC), an area known to upregulate its activity during explicit cues to synchronize to a beat^70,71^.

Furthermore, the same features of singing that underscore its therapeutic benefit to dysarthric speech may also explain the motor benefit we witnessed. In continuous voicing that occurs when singing, increases in phonation time and syllable lengthening lead to greater connectedness between words. This fluency-enhancing effect on speech may translate to motor impairments as well. As people with PD who experience vocal softness, hoarseness, and slurring when they speak are able to maintain tempo and interval variability when they sing^72^, increased vocal fluency during singing may similarly encourage motor fluidity and reduce movement variability^31^.

One limitation of this study is that we only tested one version of one song, and other musical choices might affect gait parameters differently^45,49^. Our participants had only mild-moderate disease severity, and, as external cues tend to improve gait variability for patients with greater disease progression^51^ or freezing of gait^73^, our technique should be tested on a broader spectrum of individuals. Another limitation is that all walking trials were tested on a short walkway, and some research suggests that older adults require several steps to attune to acoustic stimuli^74^ and choose different speed strategies over longer distances^75^. Although habitual walking tends to occur in short spurts, future work should explore this technique over longer distances. Lastly, as participants were never required to begin singing without hearing the song first, we do not know how this technique would translate to everyday life in which people would self-initiate their own singing. Future work should address internal cueing techniques using both beat-continuation and beat-initiation paradigms.

This study is the first to our knowledge to compare internal and external cues on walking performance in healthy adults and people with PD and to explore the effects of cueing on backward walking. While effective in laboratory settings^16–18^, external cueing has limitations that reduce its applicability to the real world. Carry-over effects are limited, so a device is required to provide constant stimulation^17,76^. Fixed-tempo rhythmic cues do not readily adapt to ever-changing environmental surroundings and are less effective than variable cues that oscillate in accordance with human gait^77–79^. Perhaps most importantly, people with PD do not report using external cues in their daily lives^35^.

Our results indicate that internal cueing through singing may be more useful than external cueing techniques for people who experience gait dysfunction from aging or neurological decline. Future work should examine different cue rates to potentially elicit stronger responses and explore rhythmic ability and musical training to elucidate who best responds to this technique. Mental singing, or singing in one’s head, should also be tested to discover if it is necessary to produce sound in order to gain benefit from singing as a cue. As external cueing is useful to a wide range of people with health conditions, from Alzheimer’s to multiple sclerosis to cerebral palsy, internal cueing may also hold benefit for myriad populations. Ultimately, a singing intervention study should be undertaken to begin to transfer this technique into a clinical setting to make it accessible to patients and carry-over effects should be tested to explore whether vocalizations enhance motor memory^61^.

## Methods

### Participants

A total of 90 participants, thirty (15 male) in each group (young control (YC), older control (OC), and Parkinson disease (PD)) took part in this study (Table 1). PD participants were recruited from the Movement Disorders Center at Washington University School of Medicine. Healthy controls were recruited via emails, social media, and flyers in and around the Washington University School of Medicine campus as well as through the Research Participant Registry through the Volunteers for Health database managed by Washington University School of Medicine. Age criteria for young controls were 18–35 whereas older controls were ≥50. PD participants were ≥50 years of age and had a neurological diagnosis of “definite PD”, as previously described^80^ and based upon established criteria^81^.

**Table 1:** Participant Demographics.

	Young control (YC)	Older control (OC)	Parkinson disease (PD)
N (male)	30 (15)	30 (15)	30 (15)
Age, yrs	25.8 (±2.8)	64.9 (±7.2)	65.8 (±6.5)
MDS-UPDRS-III	—	—	24.9 (±10.27)
MMSE, median (range)	30 (28, 30)	30 (27, 30)	29 (24, 30)
LEDD, mg	—	—	933 (±658)
Years since dx	—	—	5.77 (±3.79)
Musical experience, yrs	4.43 (±3.39)	4.42 (±6.02)	7.77 (±11.45)

Values represent mean ± SD, except where noted.

MDS-UPDRS, Movement Disorder Society Unified Parkinson Disease Rating Scale. MMSE, Mini Mental Status Examination. LEDD, Levodopa Equivalent Daily Dose.

All participants had vision corrected to 20/40 or better, were able to stand independently for at least 30 minutes, and had no evidence of dementia (MMSE ≥ 26). Participants were excluded for any history of neurological deficit (aside from PD), orthostatic hypotension, or prior deep brain stimulation surgery. One participant in the OC group was excluded for cognition as evidenced by an MMSE score of below 24 and an additional participant was recruited as a replacement.

Participants provided informed consent before participating and were compensated for their time. The protocol was approved by the Human Research Protection Office at Washington University School of Medicine, and the methods were carried out in accordance with the approved guidelines. Prior to testing, participants were assessed via the following questionnaires: the New Freezing of Gait Questionnaire (nFOGq), the Fall History questionnaire, and the Betts’ Questionnaire upon Mental Imagery (BQMI). The Movement Disorders Society Unified Parkinson’s Disease Rating Scale (MDS-UPDRS) was used to assess disease severity. Sub-sections I (non-motor symptoms), II (motor aspects of daily living), and III (motor sign severity) were administered and scored by trained staff.

### Experimental Protocol

Participants in the PD group were tested in the “on” state (i.e., they had taken their anti-Parkinson medication within the previous 2 hours) to maximize relevance to everyday walking^26^ and to optimize gait performance^82^. All walking trials were performed on a 5 m instrumented, computerized GAITRite Walkway (CIR Systems, Inc., Franklin, NJ). Three baseline trials (UNCUED) were collected in both forward and backward walking to capture each participant’s comfortable walking features. Participants then completed three walking trials in each of the conditions below in both forward and backward directions. Condition order and walking direction within each condition were randomized and counterbalanced to eliminate any training effects. In order to control cadence across conditions, participants always heard the music immediately prior to walking.

1. MUSIC: Participants listened to one verse of the song and then began walking to the beat of the song while the song looped for the duration of the walking trial. This condition is similar to a beat-synchronization paradigm and replicates traditional external cueing techniques.
2. SING: Participants listened to one verse of the song, but then the music stopped and they began singing aloud and walking to the beat of their singing. In this condition, no external source provided a cue while they walked, so participants had to generate the cue themselves.

For all cued conditions (both MUSIC and SING), we used an instrumental version of “Row, row, row your boat” that was designed with a salient beat that participants could readily detect. All participants were familiar with the melody and lyrics and sang the song without difficulty. The musical cue was administered from a laptop connected to speakers no farther than 10 m from the participant during walking and at an audible volume. Song tempo was adjusted maintaining key consistency via Audacity open source audio editing software (The Audacity Team, audacity.sourceforge.net/) to match preferred cadence in each direction, as determined from the baseline trials. Cue rate was set to 100% of preferred cadence of each direction so as not to complicate task demands, particularly for backward walking.

### Data analysis

Statistical analyses were done using IBM SPSS Statistics 24. For each participant, data were averaged across the three trials of each condition. Gait characteristics (velocity, cadence, and stride length) and variability (coefficients of variation for stride length, stride time, and single support time) were compared in two separate analyses, one for each walking direction. Normalized velocities were calculated as velocity/average leg length (cm/s/leg length) and coefficients of variation (CV) were calculated as the ((standard deviation/mean) × 100) for each person in each condition. As we were only interested in how cueing affected these measures, we ran analyses on each variable as it compared to the UNCUED condition. Hence, gait characteristics were expressed as a percent change from UNCUED and gait variabilities were expressed as a change in CV from UNCUED. Mixed model repeated measures ANOVAs with between-subject factor of group and within-subject factor of condition were used to assess differences, and Tukey-corrected post-hoc pairwise comparisons were used as appropriate. Statistical significance was set at α = 0.05.

The datasets generated and analyzed during the current study are available from the corresponding author on reasonable request.

## Electronic supplementary material

Supplementary Table 1
