# Supplementary material for: Internal cueing improves gait more than external cueing in healthy adults and people with Parkinson disease
Source: Sci Rep. 2018 Oct 19;8:15525. doi: 10.1038/s41598-018-33942-6 (PMC6195608; doi:10.1038/s41598-018-33942-6)
Supplement: Supplementary file 1 — Supplementary Table 1 [file 41598_2018_33942_MOESM1_ESM.pdf]

Internal cueing improves gait more than external cueing in healthy adults and people with Parkinson disease

Authors: Elinor C Harrison[1], Adam P Horin[1], and Gammon M Earhart[1-3,*]

[1]Program in Physical Therapy, Washington University School of Medicine, St Louis, Missouri, United States of America
[2]Department of Neurology, Washington University School of Medicine, St Louis, Missouri, United States of America
[3]Department of Neuroscience, Washington University School of Medicine, St Louis, Missouri, United States of America

Supplementary Table 1. Means and standard deviations of gait characteristics and variabilities for different cueing conditions, averaged across participants for each group.

		Normalized Velocity (cm/s/leg length)			Cadence (steps/min)			Stride Length (cm)		
		YC	OC	PD	YC	OC	PD	YC	OC	PD
**Forward**	**Uncued**	1.71 (0.21)	1.66 (0.16)	1.53 (0.21)	110.58 (6.74)	112.14 (6.02)	110.90 (7.84)	149.47 (10.31)	143.51 (15.28)	134.08 (15.58)
	**Music**	1.70 (0.20)	1.69 (0.16)	1.56 (0.20)	110.60 (7.31)	113.28 (6.66)	111.13 (7.71)	148.58 (9.29)	144.34 (12.10)	134.16 (15.66)
	**Sing**	1.73(0.22)	1.68 (0.17)	1.56 (0.26)	112.49 (7.89)	114.43 (7.62)	112.72 (8.63)	147.93 (10.16)	142.15 (13.22)	133.02 (17.23)
**Backward**	**Uncued**	1.32 (0.22)	1.17 (0.19)	0.99 (0.23)	107.29 (9.19)	110.86 (9.46)	112.30 (9.93)	118.72 (13.36)	102.77 (16.47)	83.48 (21.55)
	**Music**	1.33 (0.21)	1.26 (0.21)	1.07 (0.22)	107.24 (9.12)	110.72 (9.44)	112.96 (10.43)	119.83 (12.33)	109.67 (15.88)	90.45 (20.35)
	**Sing**	1.41 (0.21)	1.28 (0.21)	1.10 (0.22)	110.71 (8.68)	112.89 (9.54)	115.54 (10.02)	123.38 (13.14)	109.86 (17.04)	91.15 (20.90)

		Stride Length CV			Stride Time CV			Single Support Time CV		
		YC	OC	PD	YC	OC	PD	YC	OC	PD
**Forward**	**Uncued**	1.83 (0.77)	2.18 (0.67)	2.73 (1.43)	1.73 (0.59)	2.19 (0.82)	2.24 (0.71)	2.55 (0.75)	3.19 (1.07)	3.78 (1.09)
	**Music**	2.77 (1.19)	2.79 (1.32)	4.07 (2.59)	2.25 (0.85)	2.32 (1.05)	2.45 (0.77)	3.43 (1.17)	3.78 (1.77)	4.32 (1.58)
	**Sing**	2.04 (0.84)	2.35 (1.04)	2.52 (0.93)	1.65 (0.61)	1.86 (0.57)	2.02 (0.66)	3.02 (0.79)	3.18 (0.71)	3.82 (1.25)
**Backward**	**Uncued**	4.33 (1.44)	6.11 (2.38)	7.77 (2.99)	3.11 (0.97)	3.84 (1.28)	4.15 (1.54)	5.03 (1.18)	5.97 (1.68)	7.14 (2.70)
	**Music**	5.35 (2.14)	6.18 (2.35)	7.59 (2.54)	3.27 (1.89)	3.01 (0.86)	3.63 (1.89)	5.29 (2.36)	5.44 (1.79)	6.63 (2.83)
	**Sing**	4.19 (1.42)	5.65 (2.03)	7.12 (2.97)	2.81 (0.71)	2.64 (0.86)	3.25 (1.19)	4.56 (1.12)	4.99 (1.45)	5.99 (1.98)

See figures for significance.
